# The Mystery of the Borgia Poison

**Published:** 1909-08-21

**Authors:** 


### Medical Antiquities.

### THE MYSTERY OF THE BORGIA POISON.

Tradition has linked the name of Borgia with lurid stories of crime and secret poisoning mingled with the romance of the Middle Ages. Much has been written concerning this powerful family, which came into notoriety in the time of Pope Calixtus III. about the year 1455. From recent research into the chronicles of the period it would appear that some of its members were not so black as tradition has painted them, and many of the crimes attributed to them cannot be altogether proved. But few traditions are without a germ of truth, and there is little doubt that some of the Borgias were guilty of sinister deeds, which were only too common in the times in which they lived.

The family was of Spanish origin, and migrated to Italy, the first famous member being Rodrigo, who was born in 1431. He first became a soldier, but afterwards, through the influence of his relative Calixtus, entered the priesthood, and finally rose to be the head of the Church under the title of Pope Alexander VI. He had five children by Vanozza de Cattanei—namely, Pier Luigi, who died in infancy, Giovanni Duke of Gandia, Giffredo Count of Cariati, Cesare, afterwards Duke of Valentinois, and Lucrezia, who eventually became Duchess of Ferrara.

While Lucrezia was but fifteen years of age her father, then Cardinal, betrothed her to a Spanish gentleman; but on obtaining the pontificate he broke off the engagement, with the evident object of marrying his daughter to a man of higher rank, and on June 12, 1493, Lucrezia was married to Giovanni Sforza, Lord of Pesaro. The marriage was not by any means a happy one, and at the end of four years it was dissolved by the Pope, who had other motives in view, for he soon arranged a marriage between Lucrezia and Alfonso Duke of Bisceglie, a natural son of Alfonso II., King of Naples. This marriage took place in 1498. Soon after the birth of her first child, the Duke, her husband, was attacked by several men and severely wounded. Lucrezia is said to have nursed him back to health, but in her absence the Duke was murdered, it is said, at the instance of Cesare Borgia, his brother-in-law.

After her husband's death Lucrezia retired for a time to Nepi. On her return to Rome she appears to have acted as private secretary to her father the Pope, but in about twelve months' time her betrothal to Alfonso of Este, the elder son of the Duke of Ferrara, was announced.

Lucrezia has been accused of being guilty of the worst possible crimes, including that of secret poisoning, but there is practically no historical proof of the truth of these traditions. There is little doubt that many of the infamous actions of her brother Cesare were reflected upon her. On December 20, 1502, the marriage of Lucrezia with Alfonso of Este was performed by proxy, and she shortly afterwards left Rome to take up her residence in Ferrara.

In the summer of the year 1503 Pope Alexander VI., Lucrezia's father, died under mysterious circumstances, which some chroniclers attribute to poison, while others declare his death was due to a tertian ague. Muratori quotes many authorities to prove that the death of Alexander was not caused by poison, and the balance of evidence certainly seems in favour of the theory that, despite all his crimes, Alexander VI. died from a natural cause, which was probably a fever of virulent type.

It is reasonable to assume that many an individual whose death is said to have been due to poison, merely fell a victim to one of the diseases that were ever rife in the cities in the Middle Ages, which arose mainly from the insanitary conditions under which the people lived.

An inspection of many of the old Italian palaces will confirm the opinion, that typhus and other virulent forms of fever might have been readily contracted under the conditions in which their inmates lived, with a total lack of proper ventilation and ordinary sanitary necessities. It is true that many persons accused of poisoning did under torture frequently admit their guilt, but such confessions must generally be regarded as unreliable.

At the time of his father's death Cesare Borgia was sick in bed, his illness, it is said, being caused by swallowing a portion of the poisoned sweetmeats which cost his father his life. Cesare, it is related, partook of the poisoned sweetmeats in error, and omitted to carry out the advice of Macchiavelli always to carry an antidote with him. More probably he was suffering from an attack of the same fever which his father had contracted.

His death was lamented at least by one person, and that was his sister Lucrezia, who at once set out for the Monastery at the Corpo di Cristo to offer prayers for his soul, where she remained two nights.

One or two entries in the book of her household expenses are not without interest. In 1507: "To Maestro Ludovico, physician to her Highness, 110 lire for the balance of his salary." "On the 31st of December, 240 lire as a year's salary for her Highness's physician, Maestro Ludovico, at the rate of 20 lire a month." Surely a very modest stipend for a physician to a Duchess?

Patroness of poets and painters, Lucrezia made herself popular in Ferrara. In the Library of Modeno is a list of her magnificent jewels which she sold to free her husband from the debts he contracted during the wars in defence of his territories. Many of her letters, still extant, show that during these troublous times the relief of the poor, sick, and needy was Lucrezia's constant care. She died during her confinement on June 21, 1519. The accouchement had been long and difficult, and the officers and servants of her household were clustered at the foot of the grand staircase leading to her room. Great fears were entertained as to her recovery, and they waited in breathless silence for every sound from the apartment. At length Maestro Alberti, the Court Apothecary, was seen descending the staircase with a jug in his hand. All pressed forward to ask him where he was going. He replied significantly, "To get some rose-water to wash the body of the Duchess."

According to tradition, the Borgia family are credited with the knowledge of a secret poison said to be of a specially potent type, which they employed to remove from their path any who opposed them. Many stories are related of this celebrated venom, and some early writers go so far as to record the method of its preparation. According to one chronicler, it is said to have been prepared by cutting open a pig, powdering the carcass thickly with arsenic, after which it was left to decompose. The liquid collected from the putrefying mass was then collected, and formed the substance used. Another states, a bear was killed, then cut open and treated in a similar manner, and the liquid that dripped from it formed the poison.

This method of preparing a venom was very probably used by some Italian poisoners, and was known at that period. The combination of the animal poison contained in the products of putrefaction, together with arsenic, would no doubt furnish a venom of a very powerful nature, but whether the Borgia family ever used such a poison there is no evidence to prove.

In connection with the Borgia poison there is a curious story that the secret of its preparation perished with the Duc Riaro-Sforza, who died in Paris about fifty years ago. Before his death, one evening at the opera, the Duke confided to a distinguished critic, who occupied the neighbouring stall, that he still possessed the secret of the famous poison, although for centuries it had lain idle in the family archives. Its composition was, he added, simpler than was generally supposed, and not long afterwards he told his friends that, feeling age advancing and having no direct heirs, he had thought it best to burn the recipe lest it might fall into bad hands.

Baron Corvo, in his "Chronicles of the Borgias," scouts the idea that the family possessed any such secret, and denies that the venom ever existed. The probability is, that if the Borgias ever found it necessary to use a poison for nefarious purposes they employed arsenic, which was commonly used in Italy at that period. The fact that Cesare Borgia's signet ring, which is still in existence, contains a secret receptacle, which may easily have been used to carry arsenic, goes far to substantiate this conjecture, and is the strongest evidence we have that he at least used some poisonous substance to further his evil designs.

